# Characteristics of outpatient nirmatrelvir/ritonavir recipients during the 2022/2023 era in Alberta, Canada: A retrospective observational population-based study

**DOI:** 10.14745/ccdr.v52i06a06

**Published:** 2026-06-30

**Authors:** Khanh Vu, Jason Randall, Karen Martins, Sylvia Aponte-Hao, Lynora Saxinger, Jenine Leal, Elissa Rennert-May, Ellen Rafferty, Phuong Uyen Nguyen, Tyler Williamson, Scott Klarenbach

**Affiliations:** 1 Real World Evidence Unit, Faculty of Medicine and Dentistry, University of Alberta, Edmonton, AB; 2 Research Services, Alberta Strategy for Patient Oriented Research SUPPORT Unit (AbSPORU), Calgary, AB; 3 Centre for Health Informatics, Cumming School of Medicine, University of Calgary, Calgary, AB; 4 Department of Medicine, University of Alberta, Edmonton, AB; 5 Department of Microbiology and Immunology, University of Alberta, Edmonton, AB; 6 Infection Prevention & Control, Alberta Health Services, AB; 7 Department of Community Health Sciences, Cumming School of Medicine, University of Calgary, Calgary, AB; 8 Department of Microbiology, Immunology, and Infectious Diseases, Cumming School of Medicine, University of Calgary, Calgary, AB; 9 O’Brien Institute for Public Health, University of Calgary, Calgary, AB; 10 Department of Medicine, University of Calgary, Calgary, AB; 11 Institute of Health Economics, Edmonton, AB; 12 Libin Cardiovascular Institute, University of Calgary, Calgary, AB; 13 Alberta Children’s Hospital Research Institute, University of Calgary, Calgary, AB

**Keywords:** Paxlovid, COVID-19, oral antiviral therapy, outpatient use, real-world, administrative data, observational, population-based

## Abstract

**Background:**

An understanding of real-world nirmatrelvir/ritonavir (NMV-r; Paxlovid™) use in Canada is needed to inform strategies for therapy access and use.

**Objective:**

To describe the characteristics of adults who received NMV-r in Alberta, Canada.

**Methods:**

Population-level administrative data was used to describe adults (18 years and older) who received an outpatient dispensation of NMV-r between January 2022 and March 2023 in Alberta. Omicron variants predominated during this period.

**Results:**

Mean age of the cohort (n=11,793) was 65 years (standard deviation=18), 60.4% were female and 83.9% had one or more health condition associated with a risk for the development of severe COVID-19 outcomes. In comparison to the general Alberta population, a larger proportion of those that received NMV-r resided in urban areas (Alberta: 82.3%; NMV-r: 89.8%) and were more socioeconomically well-off (Material Deprivation Index quintile 1-Alberta: 20.0%; NMV-r: 25.8%). A total of 81.6% received three or more COVID-19 vaccine doses and 7.0% received one or no dose. The majority of dispensed NMV-r prescriptions were from physicians (83.6%), with 13.5% from pharmacists and 2.9% from other healthcare providers.

**Conclusion:**

In Alberta, adults who received outpatient NMV-r displayed characteristics associated with risk for progression to severe COVID-19 outcomes (related to age and health conditions) and healthcare-seeking behaviour (older age, female sex, presence of health conditions, higher socioeconomic status and highly vaccinated). Provision by urban/rural and socioeconomic status were noted. Findings can be used to inform strategies for overcoming barriers and ensuring equitable access to COVID-19 therapy for all who are most likely to benefit.

## Introduction

The COVID-19 pandemic, caused by severe acute respiratory syndrome coronavirus 2 (SARS-CoV-2), has been the most disruptive public health crisis of the 21^st^ century. While widespread immunization (which began December 2020 in Canada), immunity from previous infections and prevalence of newer variants associated with milder disease have contributed to a reduction in the risk of severe COVID-19 illness (1,2), some individuals remain at high risk, particularly older adults and those living with a higher comorbid burden and certain health conditions (3). Among these individuals, antiviral therapies for the treatment of COVID-19 and prevention of severe outcomes such as hospitalization and death are valuable tools.

Nirmatrelvir/ritonavir (NMV-r; Paxlovid™), which became available in Canada on January 18, 2022, is an oral antiviral medication for the treatment of COVID-19 in adults that can be taken within five days of mild-to-moderate symptom onset in those who test positive for SARS-CoV-2 and who are at high risk for developing severe COVID-19 outcomes (4). Due to early supply constraints, initial eligibility in Canada was limited to those who were considered to benefit most, with varied provincial criteria. As knowledge and availability of therapy increased, access to NMV-r expanded with broader eligibility criteria, and inclusion of pharmacist prescribers and nurse practitioners to provide increased access to timely treatment for COVID-19 and ease pressure on the healthcare system (**Appendix**, Supplemental material, Table S1) (5–11).

Although outpatient NMV-r therapy has been shown to significantly reduce the risk of severe COVID-19 outcomes among those at high risk (12–15), previous reports from the United States have shown that NMV-r is underused in the eligible population (16–18). An understanding of real-world population-level NMV-r prescribing and use in the outpatient setting would inform strategies to improve therapy access and use, which, in turn, could reduce serious COVID-19 outcomes and attendant strain of the healthcare system. Limited information is available in Canada. Sicard *et al.* (2023) described the characteristics of NMV-r recipients from eight jurisdictions across Canada during the first several months of its availability (19). Updated and additional information describing the demographic and clinical characteristics of this population of interest in Canada, along with healthcare prescriber type would be beneficial. The objective of this study was to describe the characteristics of adults who received an outpatient dispensation for NMV-r in Alberta, Canada between January 2022 and March 2023.

## Methods

Ethics approval was received from the University of Alberta (Pro00132799) and informed consent was waived. Data custodian approvals were received from Alberta Health and Alberta Health Services for the use of administrative health data. A retrospective, observational, population-based study was conducted using administrative health data from several databases in Alberta (Appendix, Supplemental material, Table S2).

Eligibility criteria included those who 1) were 18 years of age or older on the index NMV-r dispensation date (date of the first NMV-r dispensation during the inclusion period between January 18, 2022 to March 31, 2023; Omicron BA1.1, BA.2, BA.2.12.1, BA.4, BA.5, BQ.1, BQ.1.1 and XBB.1.5 variants predominated during this period) and 2) had provincial healthcare coverage for two or more years before the index NMV-r dispensation date. Canadian provinces have single-payer health systems and provide publicly funded medically necessary care for all residents. While the majority of prescription drugs are not publicly funded, the Public Health Agency of Canada procured, allocated and paid for NMV-r during the inclusion period of this study.

Demographic characteristics recorded on the index NMV-r dispensation date included age, sex, urban/rural residence, those residing within long-term care or supportive living and socioeconomic status (20). Clinical characteristics included the Charlson Comorbidity Index score (Appendix, Supplemental material, Table S3) (21,22) and health conditions that were eligible for NMV-r in Alberta during the inclusion period (Appendix, Supplemental material, Table S4) (7,9,21,23–39). The proportion of those who had a prior infection of SARS-CoV-2, and the previous number of COVID-19 vaccine doses received, were reported. Descriptive statistics included counts and percentages for categorical variables and means and standard deviations (SDs) for continuous variables. In accordance with data custodian privacy standards, outcomes with one to nine individuals were reported as “fewer than 10” (40); where applicable, other outcomes were censored. Analysis was performed using SAS (version 9.4; SAS Institute, Cary, North Carolina).

## Results

**Figure 1:**
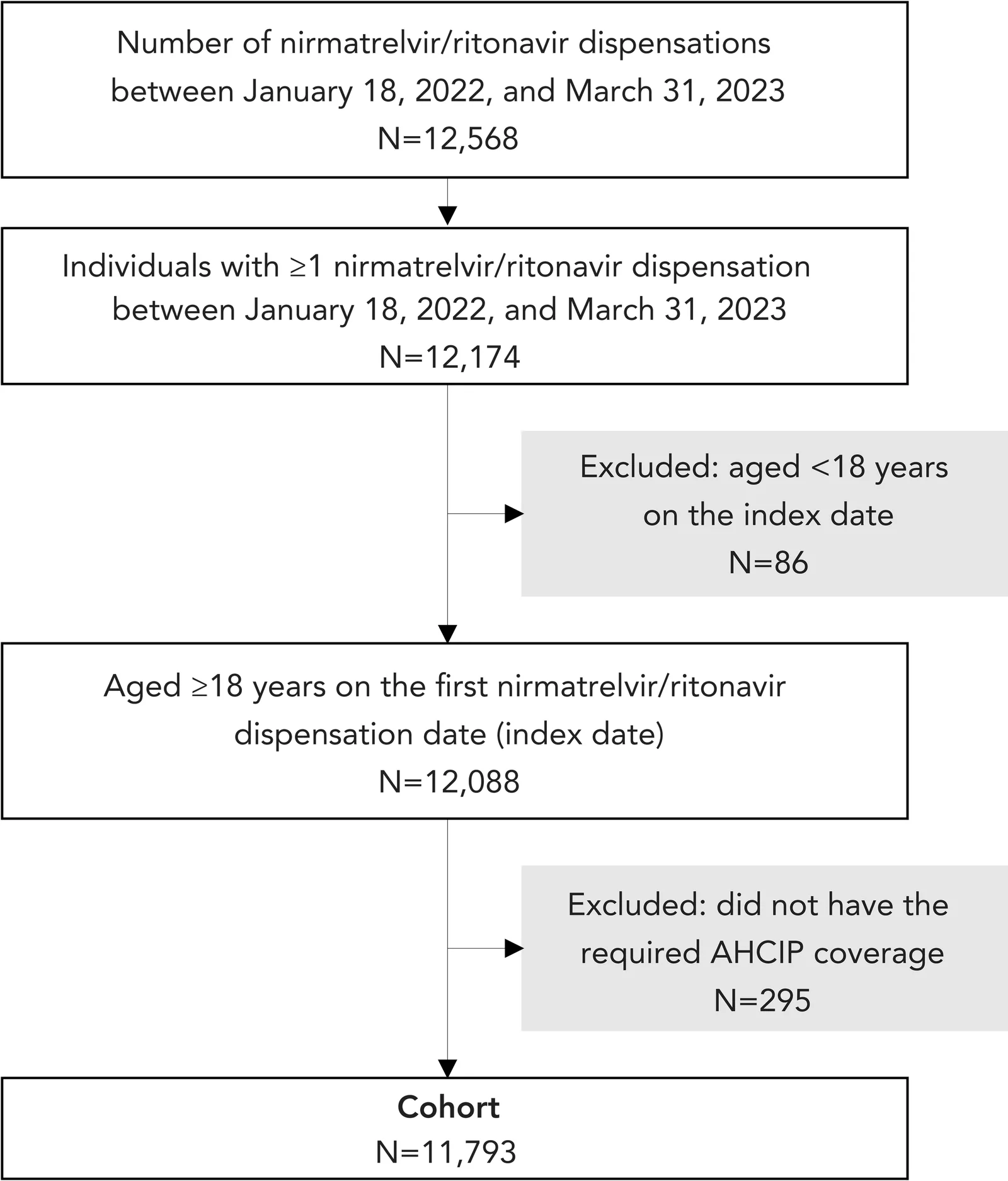
Cohort selection flow diagram Abbreviation: AHCIP, Alberta Health Care Insurance Plan

Cohort selection is shown in **Figure 1** (Appendix, Supplemental material, Figure S1 includes data linkage). Mean age of the cohort (n=11,793) was 65 years (SD=18), 60.4% were female, the majority lived in urban areas (cohort: 89.8%; Alberta general population residing in an urban area: 82.3% (41)) and 7.8% lived in long-term care or supportive living (**Table 1**). Socioeconomic status was presented based on quintiles that represent the Alberta general population (five groups with 20% in each); comparatively, the cohort was more likely to be materially well-off (Material Deprivation Index [MDI] 1: 25.8%) and less likely to be materially deprived (MDI 5: 15.1%) (Table 1). The most commonly (>10%) identified health conditions of interest that the cohort were living with were an immunocompromised status (60.0%), obesity (22.0%), chronic kidney disease (21.0%), diabetes and taking medication for the condition (17.9%), and chronic obstructive pulmonary disease (13.7%); 83.9% had ≥1 health condition of interest (**Table 2**). Most individuals received ≥3 doses of a COVID-19 vaccine on or before the index NMV-r dispensation date (81.6%), 11.4% received 2 doses, and 7.0% received ≤1 dose (0 doses: 5.9%; 1 dose: 1.1%) (Table 2).

**Table 1:** Demographic characteristics of the total cohort

Demographic characteristics	n (%) (N=11,793)
**Age, years**	
Mean (SD)	65 (18.0)
**Category**	
18–49	2,460 (20.9%)
50–59	1,636 (13.9%)
60–69	2,428 (20.6%)
70 or older	5,269 (44.7%)
**Sex**	
Female	7,127 (60.4%)
Male	4,666 (39.6%)
**Residence**	
Urban	10,589 (89.8%)
Rural	1,204 (10.2%)
Long-term care/supportive living	916 (7.8%)
**Socioeconomic status**	
**Material Deprivation Index**	
1 (most well-off)	2,824 (25.8%)
2	2,423 (22.1%)
3	2,027 (18.5%)
4	2,033 (18.5%)
5 (most deprived)	1,657 (15.1%)
Missing	829
**Social Deprivation Index**	
1 (most well-off)	2,366 (21.6%)
2	1,915 (17.5%)
3	2,044 (18.6%)
4	2,255 (20.6%)
5 (most deprived)	2,384 (21.7%)
Missing	829

Abbreviation: SD, standard deviation

**Table 2:** Clinical characteristics of the total cohort

Clinical characteristics	n (%) (N=11,793)
**Charlson Comorbidity Index**	
Overall score, mean (SD)	2.0 (2.2)
**Category**	
0; no comorbidity	3,066 (26.0%)
1–2; mild comorbidity	5,317 (45.1%)
3–4; moderate comorbidity	2,118 (18.0%)
5 or more; severe comorbidity	1,292 (11.0%)
**Health conditions**	
**Type of condition**	
Immunocompromised	7,073 (60.0%)
Obesity	2,600 (22.0%)
Chronic kidney disease	2,478 (21.0%)
Diabetes, taking medication	2,106 (17.9%)
COPD	1,613 (13.7%)
Asthma	1,000 (8.5%)
Congestive heart failure	832 (7.1%)
Pregnant	97 (0.8%)
**Number of above conditions**	
0	1,907 (16.2%)
1	4,798 (40.7%)
2	3,073 (26.1%)
3 or more	2,015 (17.1%)
**COVID-19-related characteristics**	
Prior SARS-CoV-2 infection	603 (5.1%)
**COVID-19 vaccine doses received**	
0	697 (5.9%)
1	131 (1.1%)
2	1,344 (11.4%)
3 or more	9,621 (81.6%)

Abbreviations: COPD, chronic obstructive pulmonary disorder; SARS-CoV-2, severe acute respiratory syndrome coronavirus 2; SD, standard deviation

Among those with an identified prescriber type (n=10,072; 85.4% of the cohort), 83.6% (n=8,418) had NMV-r prescribed by a physician; the most common types were primary healthcare providers (78.8%; n=6,630) and emergency medicine specialists (14.2%; n=1,197) (**Table 3**). Other providers included pharmacists (13.5%; n=1,360) and other types of healthcare providers (2.9%; n=294), of whom the majority were nurse practitioners (54.1%; n=159) and those from pulmonary function laboratories (41.5%; n=122) (Table 3).

**Table 3:** Healthcare prescriber type of outpatient pharmacy dispensed nirmatrelvir/ritonavir

Healthcare provider type	Prescriber type identified, n (%) (N=10,072)
**Physician**	**8,418 (83.6%)**
Primary healthcare provider	6,630 (78.8%)
Emergency medicine	1,197 (14.2%)
Obstetrics and gynecology	208 (2.5%)
Infectious disease	90 (1.1%)
Cardiology	78 (0.9%)
Internal medicine	57 (0.7%)
Hematology	34 (0.4%)
Mental health	33 (0.4%)
Paediatrics	17 (0.2%)
Other	fewer than 10 of each type
**Pharmacist**	**1,360 (13.5%)**
**Other types**	**294 (2.9%)**
Nurse practitioners	159 (54.1%)
Pulmonary function laboratory	122 (41.5%)
Other	fewer than 10 of each type

## Discussion

This retrospective, observational, population-based study characterised adults who received outpatient NMV-r in Alberta, Canada between January 2022 and March 2023. Recipients were generally older in age and were living with health conditions associated with a high risk for the development of severe COVID-19 outcomes. This is an important finding as individuals who are at low risk have been shown to not benefit from NMV-r use (42,43). Individuals were also highly vaccinated, with 81.6% having received three or more COVID-19 vaccine doses. There was evidence of higher use among those who resided in urban areas and in areas with a higher socioeconomic status, indicating potential urban/rural and socioeconomic inequalities. The majority of dispensed NMV-r prescriptions were from physicians (83.6%), with 13.5% from pharmacists and 2.9% from other types of healthcare providers. Findings show that while NMV-r was received by adults at high risk for progression to severe COVID-19 outcomes, strategies are needed to address barriers and ensure equitable access to COVID-19 therapy for all who are most likely to benefit.

Sicard *et al.* (2023) investigated the characteristics of NMV-r recipients in Canada during the first several months of its availability (19). The authors found that 61% of individuals were 70 years of age and older, 56% were female, 67% had one or more health conditions and 84% had received three or more COVID-19 vaccine doses, with 5% unvaccinated (19). Similar results were observed in this study regarding the proportion of those who were female and COVID-19 vaccination status; differences were observed with age and health conditions. In this study, a lower proportion who received NMV-r were 70 years of age and older (44.7%) and a higher proportion had one or more health condition (83.9%). The longer time frame over which the current study inclusion period occurred included broadening eligibility at younger ages (Appendix, Supplemental material, Table S1). The number and type of included health conditions, and the methodology used to define and identify these conditions differed between studies, likely contributing to the varying proportion of people living with health conditions of interest who received NMV-r.

Large retrospective, observational studies from the United States have shown that among individuals who were SARS-CoV-2-positive and eligible to receive NMV-r, the likelihood of receiving NMV-r increased with having a higher socioeconomic status (versus lower), being female and receiving COVID-19 vaccine doses (particularly three or more versus none) (18,44). Findings from the current study extend these previous reports by indicating urban residents may also be more likely to receive NMV-r. A number of the characteristics associated with higher use of NMV-r are consistent with healthcare-seeking behaviour, which has been shown to be more prevalent in those with a higher socioeconomic status, female sex/gender, older age, the presence of chronic conditions, knowledge of illness prevention and health maintenance, and trust in healthcare providers (45). Although not investigated in this study due to data limitations, previous reports have also shown racial and ethnic disparities in the use of outpatient NMV-r (17,46). Policies and programs addressing barriers to equitable healthcare access and COVID-19 vaccine acceptance and uptake may provide insights for initiatives to reduce outpatient COVID-19 therapy disparities (47). This is of particular importance now that the Government of Canada is no longer supplying COVID-19 rapid antigen tests and NMV-r free of charge, and coverage for these costs varies across provinces and territories (48).

Although prescribing of NMV-r was expanded to pharmacists and nurse practitioners to provide increased access in Alberta, 83.6% of dispensed NMV-r prescriptions were still from physicians. Gold *et al.* (2022) found that despite a substantial increase in the number of COVID-19 oral antiviral dispensing sites in the United States, population-adjusted dispensing rates in areas with a low socioeconomic status (versus higher) were substantially lower even though these areas had the most dispensing sites (49); therefore, addressing outpatient COVID-19 therapy disparities among high risk individuals will require initiatives beyond pharmacist prescribing and pharmacy proximity.

Although the clinical profile of individuals at high risk for severe COVID-19 outcomes has evolved since the inclusion period of this study, data support NMV-r as an ongoing first-line treatment (14,15). Consequently, addressing disparities in outpatient treatment access among this population remains a relevant need. Current guidelines suggest outpatient NMV-r treatment for those with older age, immunocompromising conditions, or multiple comorbidities, regardless of vaccination status; particularly, adults aged 75 years and older, adults of any age living with an immunocompromising condition, and adults who are immunocompetent living with multiple risk factors for progression to severe COVID-19 illness (e.g., aged 65 years and older, medical complexity, certain health conditions) (3).

### Limitations

This study has several important strengths including the large size and population-based design; however, this study is also subject to limitations that should be taken into consideration when interpreting results. Retrospective claims-based studies use administrative data with consequent potential for misclassification of study cohorts or measures; validated case definitions were used, where available, to address this limitation. Socioeconomic status was determined at the neighbourhood dissemination area level (a small geographic area), as opposed to the individual living within the neighbourhood; therefore, there is a potential for misclassification of some participants within the quintiles. Race and ethnicity are not included in administrative health data and therefore could not be assessed. The Pharmaceutical Information Network database only provides information on prescription medication dispensations, and, therefore, does includes neither medications prescribed but not dispensed nor uptake by individuals.

## Conclusion

Results from this real-world study showed that adults who received outpatient NMV-r in Alberta displayed characteristics consistent with a high risk for progression to severe COVID-19 outcomes (related to age and health conditions) and healthcare-seeking behaviour (older age, female sex, presence of health conditions, higher socioeconomic status and highly vaccinated). Results also suggested potential urban/rural and socioeconomic differences.

Achieving the optimal use of NMV-r will require iterative refinement of high risk criteria to reflect the evolving epidemic and evidence of benefit, ensuring high risk groups can access diagnostic testing, along with addressing barriers such as drug costs, knowledge gaps and inequities. The implementation of initiatives should be paired with assessment to confirm their effectiveness. If risk-based deployment of NMV-r is successful and barriers are overcome, serious COVID-19 outcomes and attendant strain of the healthcare system could be reduced.

## Appendix

Supplemental material is available upon request to the author: swk@ualberta.ca

Table S1: Eligibility criteria for nirmatrelvir/ritonavir in Alberta during the study inclusion period

Table S2: Administrative databases used in the study

Table S3: Health conditions and their associated codes and weights included in the Charlson Comorbidity Index

Table S4: Case definitions used for identification of health conditions

Figure S1: Selection of the study cohort with data linkage shown
